# Does AI Debias Recruitment? Race, Gender, and AI’s “Eradication of Difference”

**DOI:** 10.1007/s13347-022-00543-1

**Published:** 2022-10-10

**Authors:** Eleanor Drage, Kerry Mackereth

**Affiliations:** Centre for Gender Studies and the Leverhulme Centre for the Future of Intelligence, University of Cambridge, Cambridge, UK

**Keywords:** Artificial intelligence, Recruitment, Hiring, Bias, Race, Gender

## Abstract

In this paper, we analyze two key claims offered by recruitment AI companies in relation to the development and deployment of AI-powered HR tools: (1) recruitment AI can objectively assess candidates by removing gender and race from their systems, and (2) this removal of gender and race will make recruitment fairer, help customers attain their DEI goals, and lay the foundations for a truly meritocratic culture to thrive within an organization. We argue that these claims are misleading for four reasons: First, attempts to “strip” gender and race from AI systems often misunderstand what gender and race are, casting them as isolatable attributes rather than broader systems of power. Second, the attempted outsourcing of “diversity work” to AI-powered hiring tools may unintentionally entrench cultures of inequality and discrimination by failing to address the systemic problems within organizations. Third, AI hiring tools’ supposedly neutral assessment of candidates’ traits belie the power relationship between the observer and the observed. Specifically, the racialized history of character analysis and its associated processes of classification and categorization play into longer histories of taxonomical sorting and reflect the current demands and desires of the job market, even when not explicitly conducted along the lines of gender and race. Fourth, recruitment AI tools help produce the “ideal candidate” that they supposedly identify through by constructing associations between words and people’s bodies. From these four conclusions outlined above, we offer three key recommendations to AI HR firms, their customers, and policy makers going forward.

## Introduction

In mid-April 2020, only months into the COVID-19 pandemic, a Gartner, Inc. poll of 334 HR leaders found that 86% of organizations were incorporating new virtual technology into their hiring practices (Gartner, 2020). With lockdown restrictions set to persist, artificial intelligence (AI) uptake rapidly increasing across all domains, and recruitment practitioners dissatisfied with traditional methods, it is unsurprising that HireVue noted a 614% increase in their Japanese clients’ AI hiring activity (HireVue n.d.). While HireVue no longer uses AI in its video hiring software following an external audit, other companies are stepping in to fill its shoes, notably Retorio and myInterview, founded in 2018 and 2016 respectively. These AI-powered human resources (HR) video tools claim to simultaneously streamline and debias recruitment. In doing so, they promise to solve two key challenges HR professionals currently face: first, workload increases prompted by high volume recruitment; and second, the pressure to fulfill corporate diversity, equality, and inclusion (DEI) goals. We specifically focus on Retorio and myInterview as two prominent AI video hiring tools, as well as HireVue, which now uses AI-powered keyword analysis based on its retired video hiring AI.

In this paper, we question these companies’ claims that their products are able to debias the hiring process and in doing so diversify their clients’ workforces. We argue that anti-bias measures in recruitment AI technologies reveal a prevailing misunderstanding of what race and gender are and whether they can be defined as isolatable and removable attributes within hiring. By implying that their products are able to eradicate gender and race from the hiring process, recruitment AI companies obscure how their tools operate within gendered and racialized relations of power. This evasion hinders attempts to solve issues of bias in hiring tools and poses significant risks to groups and communities who are already disproportionately at risk of experiencing negative effects from exposure to AI. We thus need a different understanding of how recruitment AI figures within systems of race and gender through their construction of ideal candidate profiles and in their readings of candidates’ voices and faces.

Despite increasing public awareness of AI recruitment tools and some attempts at regulation, AI hiring tools have received relatively little scrutiny in academic literature from the AI ethics community compared to other forms of AI development and deployment. This is surprising given the increasing prevalence of AI-powered hiring tools and the insights that they provide for thinking about the social and political impact of AI more broadly. While there are some exceptions (O’Neil, 2017; van den Broek et al. 2019; Fritts & Cabrera, 2021; Bankins, 2021; Yam & Skorburg, 2021; Ajunwa, 2021), the majority of these analyses are not grounded in the knowledge of marginalized groups and communities, for example, insights from gender and critical race theory (for an exception see Tilmes 2022 on a disability justice approach to hiring AI). This is potentially why they largely fail to interrogate a number of crucial questions relating to the ethical use of hiring AI, including what constitutes a “functioning” hiring AI system, whether they live up to their claims to strategically remove bias from the hiring process, and how their use actually affects marginalized people and communities.^1^ While we acknowledge that there are examples of the use of recruitment AI that have produced harmful outputs that disproportionately affect specific groups, such as the notorious case of Amazon’s sex-discriminatory hiring tool (Dastin, 2018), this paper does not focus on the potential discriminatory outcomes that could arise from the widespread deployment of recruitment AI. We aim to move the debate beyond whether or not AI hiring tools are “good” or “bad.” We believe that the debate over whether or not these tools are intrinsically racist or sexist, while important, may also inhibit our ability to meaningfully engage with how gender and race figure and are configured within these systems. Hence, we focus on how the producers of these technologies misunderstand how race and gender emerge and are shaped by the hiring process.

Through our analysis of recruitment AI, we contribute to a growing body of literature on AI ethics and algorithmic bias that foregrounds how AI shapes and is shaped by gender and race, as well as how gender and race are produced in the daily operations of AI systems (Weber & Bath, 2007; Strengers & Kennedy, 2020). Specifically, we enrich a growing body of feminist approaches to AI that examines how AI systems (re)produce gendered and racialized biases (Gumbus & Grodzinsky, 2004; Wellner & Rothman, 2020; Young et al., 2021; Heinrichs, 2021), while simultaneously challenging the term “bias,” calling for a greater focus on how power operates in the AI industry (Amoore, 2020; D’Ignazio & Klein, 2020; Kalluri, 2020). This means considering how, as Jason Edward Lewis notes, “The bias in these systems is not a bug but rather a feature of an interlocking set of knowledge systems designed over centuries to benefit white men first and foremost” (Lewis, 2021). Proponents of this shift from bias to power focus on how search engines are imbricated in systems of race and can therefore perpetuate racism (Noble, 2018); how AI is shaped by colonialism and the subsequent need for a decolonial approach to AI (Mohamed et al., 2020); how racial hierarchies shape the way that AI is illustrated and imagined (Cave & Dihal, 2020); and how AI perpetuates an unjust criminal justice system and dramatically expands carceral regimes into spheres of intimate and daily life (Ferguson, 2017; Wang, 2018; Benjamin, 2019a, 2019b; Schwerzmann, 2021). We push forward this body of scholarship by showing how the rapid growth of the field of recruitment AI fails to meaningfully reckon with what race and gender are and how they are embedded in recruitment AI systems, which holds serious implications for the anti-bias claims of these technologies.

The structure of this paper is as follows. We first provide an overview of the rapid uptake of AI-powered hiring tools by the HR industry. We then introduce our theoretical framework, before explaining our selection of AI recruitment firms and advertising materials. We finally report on the results and discuss the findings from our analysis, focusing on four key arguments. First, we argue that attempts made by recruitment AI companies to debias their products by stripping race and gender from their systems demonstrate a fundamental misunderstanding of what race and gender are. Second, we show how hiring AI companies’ claim to resolve some of their clients’ diversity, equality, and inclusion (DEI) issues represents a technosolutionist approach to the problem of underrepresentation in corporate contexts. Third, we explore the power relationship between the observer and the observed, examining how recruitment AI technologies reproduce histories of taxonomizing and classification. Fourth, we argue that rather than providing an objective reading of a candidate’s profile and data points, AI hiring technologies actively produce the “ideal candidate” they supposedly identify. We conclude with three recommendations as to how public and private stakeholders in the AI recruitment industry should grapple with issues of difference and discrimination that arise from the use of AI-powered tools in recruitment.

## Context

The field of hiring AI has become an increasingly significant area of AI development and deployment. A study of HR professionals representing 500 mid-sized organizations from a range of industries in five different countries found that 24% of businesses have already implemented AI for recruitment purposes and 56% of hiring managers plan to adopt it in the next year (Sage, 2020). Many HR professionals emphasize the utility of AI for increasing the efficiency of volume recruitment and workplace management (enablex 2021), to the extent that the practitioner literature on AI-powered HR tools is often “overly optimistic and paints a picture that is almost too positive” (Albert, 2019). However, as public awareness of algorithmic bias has developed, AI-powered HR tools have been subject to greater public scrutiny. In 2018, Amazon announced that they were abandoning the development of an AI-powered recruitment engine because it identified proxies for gender on candidates’ CVs and used them to discriminate against female applicants (Dastin, 2018). Meanwhile, a 2021 independent audit of Facebook’s job advertisement algorithm showed that it delivered different advertisements to male and female users based on the gendered distribution of women and men in these fields (Hao, 2021).

This heightened awareness of the use of AI tools in HR has produced some initial attempts at regulation. On January 1st 2020, Illinois’ Artificial Intelligence Video Interview Act came into effect, requiring companies that use AI to assess candidates’ “fitness” for a job position to outline what “general types of characteristics” the AI is using to evaluate and select candidates (Heilweil, 2020). Currently, the draft European Union (EU) AI Act classes AI-powered hiring software and employee management systems as “high risk” (EUR-Lex, 2021). Under the draft regulations, high-risk systems must meet a large number of requirements to be deployed in the EU, and if they do not comply, the Member State concerned must restrict, prohibit, or recall the system (EUR-Lex, 2021). The classification of HR systems as “high risk” is a promising acknowledgement of the profound effects that AI-powered HR tools could and will have on employment and the workforce. However, exactly what these obligations entail has not yet been defined.

## Theoretical Framework

In this paper, we use gender studies and critical race theory to interrogate some of the claims made by recruitment AI companies about how their tools mitigate bias. We approach gender and race as dynamic and intersecting systems of power, rather than fixed or isolatable attributes of individual bodies. While we recognize that AI-powered hiring tools will disproportionately affect a number of marginalized groups, we primarily focus on the entwined power relations of gender and race. We hope, though, that our analysis of these technologies will open up a larger discussion on recruitment AI and these tools’ relationship to social and political inequality.

Feminist and critical race scholarship have a long history of engaging productively with how “differences” emerge between groups and are sometimes named “race” and “gender,” questions of utmost relevance to recruitment AI’s attempts to debias the hiring process. We refer to the differentiation of people within social hierarchies by virtue of perceived racial and gender attributes as “racial and gender difference” for the remainder of this paper. Feminist and anti-racist theory can help inform recruitment AI design and deployment through its persuasive critique of normative universal modes of representation, taxonomization, and classification. We take particular inspiration here from scholarship on how gender is produced, perceived, and performed, such as Judith Butler’s theory of performativity and its elaboration by new materialists in science and technology studies (STS). This finding has been used by scholars to argue that AI is not observational but performative: it does not “identify” or “recognise” but merely effectuates an annotation or commentary of the bodies that emerge through AI systems (Drage & Frabetti, forthcoming). Drawing on Butler’s theory of “normative citationality”—how social norms (socially enforced rules or expectations) are repeatedly “cited”—and Karen Barad’s performative understanding of “how matter comes to matter” (both in the sense of how the material world is made meaningful and how it is made tangible), Drage and Frabetti demonstrate how AI is an observational apparatus whose production of gendered and racialized bodies always refers to and re-animates a previous source (forthcoming). This lineage of feminist scholarship allows us to interrogate how recruitment AI does not identify the attributes of job applicants, but rather plays an active role in influencing how these traits emerge and are made legible to employers.

Moreover, we work from the theoretical premise that the *denial* of difference functions as a mechanism of power. As bell hooks emphasizes, the utopian suggestion that “we” are “all the same,” that “we are all just people” is invested in the perpetuation of the status quo (1992, 167). Paul Gilroy has suggested that politically useful claims to universal humanity have been made, pointing specifically to abolitionist texts. In these texts, ideas around human “universality” formed the basis of radical social change. However, they were intended to make racism visible rather than be used as a convenient excuse not to have to engage with how systems of racialization create differences between bodies (Gilroy, 2009, 8). Drawing from hooks and Gilroy’s insights, we examine how recruitment AI’s claims to debias the hiring process functions as a denial, rather than an actual erasure, of the material and structural differences faced by individual job candidates.

We also draw on feminist and critical race theories of diversity to show how recruitment AIs can incorporate difference without bringing about necessary structural changes to the recruitment process, and in doing so reproduce an unjust status quo. Feminist and anti-racist scholars including Chandra Talpade Mohanty, M. Jacqui Alexander, Sara Ahmed, Rinaldo Walcott, and Pamela Newkirk have extensively critiqued how institutions leverage the concept of diversity in a way that supports corporate interests while simultaneously circumventing meaningful systemic change (Mohanty, 2003; Alexander, 2005; Ahmed, 2012; Newkirk, 2019). Rather than identifying and addressing structural problems, the rhetoric of diversity and unconscious bias can ultimately mask responsibility and circumvent accountability for racist and sexist harms. Through this work, we want to draw attention to how companies who develop hiring AI products use appeals to diversity as a way to market their products as both more efficient and more ethical than human recruiters.

## Methodology

### Choice of Materials

To understand how AI hiring companies approach questions of difference, diversity, and discrimination, we examine marketing and promotional materials as well as statements made by company representatives regarding how their products mitigate bias in the hiring process. Despite not engaging with the technological functioning of these tools—the algorithms of which are largely proprietary—these materials are indicative of the companies’ approaches towards race and gender bias, which is the focus of our analysis. We are interested in how promotional materials expose the interplay between corporate DEI concerns, public hype around the capabilities of AI tools, an increasingly time- and resource-pressured recruitment landscape, and a lack of public and corporate knowledge around what race and gender are and how they emerge with and through both technology and recruitment more broadly.

In the context of film and video, production culture scholar John T. Caldwell views promotional materials as “deep texts” which communicate the symbolic meaning of the advertised product (2008, 133, 405). Scholars such as Ethan Tussey have adopted Caldwell’s theories as part of qualitative and critical analyses of promotional materials in Big Tech, as Tussey has done in relation to Netflix services and other entertainment services available on portable devices (2020). In our study, an analysis of promotional materials offers a window into hiring AI companies’ perception of the problem domain—namely the recruitment industry’s concerns and priorities—and how these problems relate to the appeal of their tools.

Marketing has always had an important function in the commercialization of AI. However, there are no existing studies of AI hiring tools’ promotional materials in relation to their claims to reduce bias in recruitment. Of the online marketing tools available to AI hiring companies, websites and white papers are the most popular. White papers, which have long been a B2B marketing staple (Futurity Media, 2020), should in principle offer authoritative and in-depth research on a subject without endorsing a particular product (The University of Arizona. n.d.). However, their use by hiring AI tools is transparently product promotion, often through recourse to the language of science to substantiate their claims. We suggest that the combination of the simplification of complex technology, allusion to science, and product endorsement, when framed against the prevalent hype around AI in the recruitment landscape, can result in a miscommunication of a product’s capabilities and limitations. While Sect. 3.3 of the ASA’s Misleading Advertising CAP code demands that marketing communications do not mislead the consumer by omitting material information (ASA, n.d., 16), this becomes challenging to regulate when, as stated above, little research has been conducted into how efficacious AI hiring systems are in making companies more diverse.

The amount of data in the chosen online promotional materials varied across the different recruitment AI companies. Some websites for these products were very well developed, with those of Retorio and HireVue comprising 599 and 579 pages respectively, and myInterview’s at 97 pages. Additionally, all sites and their adjacent blogs and white papers attempt in varying degrees to explain how machine learning is used to power their tools’ functionality (Retorio, n.d., “Why Retorio”; myInterview, n.d., “myInterview intelligence”; HireVue, n.d., “Train, validate, retrain”). Retorio has 7 white papers available to download directly from their website, and HireVue and myInterview have one each, available via an email with a download link. These documents and webpages were scanned for the following keywords: “bias,” “diversity,” “race,” and “gender,” and the resulting materials formed the basis of this analysis. After focusing on webpages and materials that discuss bias, diversity, race, and/or gender, we conducted a critical discourse analysis on these texts to see how AI hiring companies were deploying these four key terms; how they engaged with the problem of bias and associated issues such as the use of proxies to discern a candidate’s protected characteristics; and how these companies propose their AI tools as a means to overcome these issues. From this discursive analysis, we identified four key claims, which we will discuss in detail below (Sects. 5.1 and 5.2).

### Selection of Hiring Tools

We conducted an initial study of video AI tools that claimed to use the Big 5 psychological assessment^2^ to reduce bias in the hiring process. From these tools, we found that myInterview, Retorio, and HireVue provide the most information about how to reduce bias through AI-powered video or transcription analysis that maps the Big 5 psychological assessment onto a candidate’s body or recorded responses. Video tools were chosen because the employment interview is one of the most common hiring practices (Roth & Huffcutt, 2013), and was greatly impacted by the COVID-19 pandemic. Furthermore, facial recognition technology (FRT) and emotion recognition technology (ERT), which play a central role in AI-powered video hiring software, are among the more controversial forms of AI, with critics suggesting that these tools have no meaningful scientific basis (Whittaker et al., 2018). This has led to heightened scrutiny of video-based AI recruitment tools, as evidenced by the passing of the 2020 Artificial Intelligence Video Interview Act in Illinois. Although, as stated above, HireVue no longer uses video software, we use HireVue as a case study due to its outsized presence in the field of recruitment: HireVue is the longest running company in the recruitment AI market and, until recently, was the most established video-based AI hiring tool. We also make reference to non-video workforce management and recruitment AI that primarily market themselves as anti-bias tools, such as Censia, in order to support our analysis of dominant anti-bias strategies in hiring AI.

Furthermore, HireVue, Retorio, and myInterview were chosen for the negative press they received debunking some of the claims they have made about their systems. For example, Bavarian Broadcasting in Munich found that Retorio’s video AI service’s perception of the applicant’s Big 5 personality can be altered by what the candidate is wearing (Fergus, 2021). Similarly, MIT Technology Review journalists found that taking myInterview’s tests in different languages changed the tool’s perception of the applicant’s personality (Schellmann & Wall, 2021). HireVue, for their part, received media attention in response to the passing of the Illinois Act, and also for their choice to disband their video service (Heilweil, 2020).

While HireVue, Retorio, and myInterview are among the most prominent AI-powered hiring tools, we also selected these tools because we believe that they are broadly emblematic of the approach that the field of AI hiring takes towards gender, race, diversity, and bias. A scoping investigation of further video hiring tools, including those which featured on Tech Target’s 2020 list of the “Top Ten Video Interviewing Tools for a Virtual Hiring Toolkit,” suggests that the findings discussed in Sects. 5.1 and 5.2 are likely to hold relatively consistently across a variety of recruitment AI tools (Feffer, 2020). Of these ten tools, five claim that their tools actively minimize or reduce bias in the hiring process without providing evidence (Arya, Zappyhire, Harquen, ModernHire, VidCruiter). Furthermore, as we will discuss in Sect. 5.2.1, an additional company, Interviewer.ai, claims that its tool is “blind to gender, ethnicity and age” when scoring candidate videos (Interviewer.ai). Hence, this first in-depth study of three video hiring tools provides a solid foundation for a further investigation of AI-powered HR software’s claims around gender, race, diversity, and bias.

## Results and Discussion

### Results

In our survey of the marketing materials offered by AI hiring firms, we identified four key claims made by AI hiring companies: (1) their tools remove bias from the hiring process, providing a more objective and neutral assessment of candidates; (2) AI-powered hiring will promote the recruitment of a more diverse workforce; (3) AI recruitment tools can identify a candidate’s internal characteristics from their external appearance; and (4) this process of categorization and sorting allows AI recruitment tools to identify a company’s ideal candidate. We discuss these claims and their implications below.

### Discussion

#### Removing Bias from the Hiring Process

One of the driving factors behind recruitment AI is its automation of purportedly “objective” forms of assessment. AI hiring firms aim to provide employers with a bias-free way of speedily processing vast numbers of applicants. The widespread uptake of AI-powered hiring technologies reflects the broader interest in AI tools as a means of addressing and rectifying implicit human biases (see Lin et al., 2021). For example, the AI hiring firm Retorio claims to provide “debiased models” that are “blind to age, gender, or skin color. We ensure that our models remove human biases and discrimination tendencies” (Retorio, n.d., “The Science Behind”). They further claim that the use of AI can help bypass the biases of human managers (Retorio, n.d. “White Paper,” 21): “AI highlights and recommends adding, removing, or replacing job board wording with neutral terms, or any wording that may lead to biased judgments, like indicators of age, gender, or ethnicity” (Retorio, n.d. “White Paper,” 21). Companies like Retorio use Big 5 psychometric testing schema because they seek to avoid forms of assessment that draw on or deploy gendered or racialized concepts. This is grounded in the assumption that the Big 5 provides a neutral assessment of a candidate’s aptitude and suitability for a job. myInterview claims that by generating an AI-powered “Personality Summary” for each candidate and “showing where they fall on each of the Big 5 personality traits” (myInterview, n.d. “myInterview Intelligence”), assessments can avoid unfairly discriminating against candidates. They reason that this is possible because their product analyzes “personality” while removing gendered and racialized personal information: “Each video is analyzed for soft skills, personality traits, and keywords so you can tell which candidates match your company vibe, while reducing the risk of bias from entering the picture” (myInterview, n.d. “Homepage”). The implication of myInterview’s claim is that the system is unbiased because it *only* sees personality, skills, and keywords, which apparently cannot be gendered or racialized. Race and gender, therefore, allegedly have little to nothing to do with how personality and suitability are assessed.

Furthermore, hiring AI companies frequently assert that they have taken the necessary technical precautions to fully strip gender, race, and other characteristics out of candidate data. On its website, Retorio details its debiasing process to make sure people of different genders and “ethnical groups” [sic] are treated fairly in the recruitment process:

In order to avoid bias and generate representative results, our datasets represent behaviors and perceptions of people from diverse populations [...] For example, in our datasets, we compare mean scores of Big 5 estimations between caucasians and blacks, young and old people, men and women, etc. If we would discover significant mean differences attributable to group membership, we would adjust the mean values and distributions to adjust for discriminatory bias in our training and test sets [...] Our findings clearly indicate that Retorio evaluates applicants regardless of their skin color, gender, or age (Retorio, n.d., “The Science Behind”).

Similarly, the AI-powered talent intelligence platform Censia aims to reduce bias the hiring process through its “anonymous mode”, which “hides names, emails, profile links, gender, and other race identifiers for an anonymized pool of candidates” (Guerrero, 2021). In an interview, CEO Jo Riley said that this mode “takes away any of those [bias] identifiers, not just stripping [down] the backend when we do a match but stripping down the front end” (What the HR!, 2020, 32.00–33.00). Censia's marketing team recognises that “Our Anonymous Mode feature isn’t a silver bullet and it can’t eliminate bias” (Guerrero, 2021). However, the functioning of its anonymous mode - which allows recruiters to toggle between a fully fleshed out and identified candidate and an anonymised ’blank slate’ - suggests that race and gender (along with other social markers) can simply be switched on and off at will in the recruitment process. Both Retorio and Censia’s promotional materials suggest that a candidate’s gender and race can, in effect, be erased within the system. This, alongside Retorio’s obsolete and inaccurate language of “caucasians and blacks,” denotes a profound misunderstanding of what systems of race and gender are and how they inform the hiring process. In all languages, Caucasian is an obsolete term that entered common parlance through the practice of scientific racism; in its first usages, it referred to people living in the Caucasus region, and soon after became mythologized in imagined racialized craniometric differences (Popejoy, 2021). Its use here is both inaccurate and carries with it racist connotations (Popejoy, 2021). Likewise, in the English language, the term “blacks” is similarly decried as an inaccurate and offensive descriptor of the Black community.

These AI hiring firms also suggest that if race and gender are simply “removed” from the equation, and therefore bias is made impossible, the best and most suitable candidates will succeed. In this sense, AI-powered hiring tools evoke a specific sociotechnical imaginary centered on the concept of meritocracy. By sociotechnical imaginary, we mean a “collectively imagined forms of social life and social order reflected in the design and fulfillment of nation-specific scientific and/or technological projects” (Jasanoff & Kim, 2009, 120). AI-powered hiring tools promise a truly meritocratic system of recruitment, where candidates are selected only on the basis of their individual characteristics and their capacity to thrive within an organization. For example, Hirevue claims that while “unconscious bias compounds at every step of the traditional hiring process[,] HireVue’s AI-driven approach mitigates bias by eliminating unreliable and inconsistent variables” (HireVue, n.d. “Increase Diversity and Mitigate Bias”). Similarly, in HireVue’s case study of how Unilever uses their software, they quote a statement made by Melissa Gee Kee, Strategy Director to CHRO and Global HR4HR Director, in which she claims that “we were hiring based solely on experience and we wanted to start screening for potential too” (HireVue, n.d. “Unilever + HireVue”). Looking at personality alone and screening for potential over other characteristics plays into both the colorblind logic of AI hiring technologies and the meritocratic emphasis on innate potential or talent as the precondition for success.

Yet even while these tools claim to neutralize markers of racial and gender characteristics in both applicants and job adverts, they also continue to market themselves through reference to racialized and gendered characteristics. For example, Censia notes that using its anonymous mode disables its “Gender diversity” filter, suggesting that recruiters can toggle at will between an ’anonymized’ candidate who will be fairly assessed and a fully fleshed-out candidate who fulfils a company’s desired diversity criteria. In this convenient logic, race and gender disappear and reappear on demand according to the needs of the recruiter. Similarly, the Retorio website features a GIF of an “applicant” in one of their interviews. She is called “Cynthia Tori,” and like the other applicants on the page is likely to have been chosen for her racially “ambiguous” appearance (in that her racial identity may be unclear to observers or signify different identities in different contexts). She also states that she likes “meeting people from different cultures” (Retorio, “Personality Assessment”). myInterview’s gesture towards multiculturalism and multiracialism epitomizes many hiring tools’ approach to gendered, racial, and cultural difference. The selection of an ambiguously racialized candidate draws on social and political discourses that romanticize mixed race and ambiguously racialized people as the bearers of a colorblind “post-racial” future (Mahtani, 2014). myInterview’s use of a multiracial avatar reflects both the futuristic and colorblind ideologies attached to mixed-race people, and the commodification of these ideas within a global capitalist marketplace (King-O’Riain and Small, 2014, viii). Retorio’s bypassing of race and national borders further evokes the sociotechnical imaginary of meritocracy through its post-racial worldview. From this perspective, multiraciality and cosmopolitanism are panaceas for deep-seated structural inequalities and political problems (Yao, 2021, 202). This logic is consistent with Retorio’s attempt to use the Big 5 to move “beyond” race, and in doing so, bring about the “pure meritocracy” that recruiters desire.

The implicit claim here is that gender and race are merely overlaid onto bodies which, “underneath it all,” are just human. While the attempt to remove race and gender from the hiring process has at its goal the possibility of assessing all humans equally, it overlooks the fact that historically the archetypal candidate has been perceived to be white and/or male and European (Todorov, 2017, 48)—a legacy which rears its head in contemporary recruitment for certain sectors, such as politics (Durose et al., 2011, 7) As feminist critics of the concept of “diversity” have noted, diversity discourses position women, queer people, and people of color as divergences from the white male norm (Ahmed, 2012). In doing so, they reposition white men as the standard bearer for what it means to be human. The desire to remove difference (and in doing so, address social inequality) corresponds to bell hooks’ account of how her students preferred to emphasize the common humanity of all people, promoting ideas of sameness over difference. hooks explained that,

Often their [the students’] rage erupts because they believe that all ways of looking that highlight difference subvert the liberal belief in a universal subjectivity that they think will make racism disappear. They have a deep emotional investment in the myth of ‘sameness’, even as their actions reflect the primacy of whiteness as a sign informing who they are and how they think (hooks, 1992, 167).

Tools like myInterview, Retorio, and Censia demonstrate this investment in sameness that hooks critiques when creating an HR system that is devoid of the discomfort of race and gender. However, it is more likely that this approach makes it easier to overlook the hiring considerations that particular groups may need in order to be assessed fairly. Moreover, as we explore in the following section on diversity, we would also caution against reducing structural discrimination to the decisions made by individual recruiters, as this is effectively a mode of escape from the real work needed to make hiring inclusive.

### Promoting Diversity Through AI Hiring Tools

From our analysis of marketing materials, it is clear that recruitment AI companies are responding to businesses’ increased emphasis on diversity, equality, and inclusion goals as corporate priorities. Retorio notes that since “85% of talent acquisition leaders feel the pressure to increase diversity […] as the use of ai [sic] to increase diversity and inclusion are reportedly working in the recruiting scene, the use of ai powered tools is continuing to grow and help recruiters during their recruitment process, as company efforts seek to reduce hiring bias” (Briah, 2021a, 2021b, “How AI is Revolutionising the Hiring Process”). Similarly, in the Carlyle group’s case studies of Unilever and the Co-Operative Bank’s use of HireVue software, HireVue emphasizes how their tools helped the Co-Operative Bank “bring their values of inclusivity to life” in response to the demand for “greater diversity and fairer hiring” (HireVue, n.d., “The Co-Operative Bank”). AI hiring firms argue that their tools can increase the diversity of a firm’s incoming workforce in two ways. First, companies such as HireVue suggest that since AI-powered hiring tools can process far larger numbers of applications than human recruiters, it also allows companies to assess a more *diverse* range of candidates: “the more talent you can interview, the greater the opportunity to diversify your candidate pool and find high-quality candidates from all backgrounds” (HireVue, n.d. “Increase Diversity and Mitigate Bias”). Secondly, AI hiring firms like myInterview and Retorio insist that the removal of bias from the hiring process will naturally result in a more diverse workforce. myInterview argues that their tools help customers to “seek diversity,” arguing that “by concentrating only on what a candidate is saying (and not how they look when they say it), our Machine Learning ensures that automated shortlisting focuses on personality to encourage diversity in your candidate pool” (myInterview, n.d., “Diverse Talent and Inclusive Talent”). The underlying message, as we have stated above, is that these tools can make meritocracy happen, and that this will necessarily result in a more diverse workforce.

However, the attempt to outsource “diversity work” to AI-powered hiring tools is also a manifestation of technosolutionism, an ideology that encourages individuals and organizations to solve social and political problems through technological innovation (Atanasoski & Vora, 2019). While we do not deny that AI-powered tools may help HR professionals recruit more diverse workforces, we also caution that such “diversity tools” may obscure the structural issues within organizations that lead to underrepresentation in workforces and exclusive work cultures. For example, in STEM industries, the terms “pipeline problem” or “leaky pipeline” are used to describe the underrepresentation of women and people of color at entry level jobs and their diminishing numbers at higher levels of employment, resulting in almost no representation at leadership level (West, Whittaker & Crawford, 2019). The pipeline problem identifies some key drivers behind the underrepresentation of women and people of color in STEM, such as the low numbers of women and girls studying STEM subjects at secondary school and at college or university (PWC, n.d. “Women in Tech,” 11). However, as West, Whittaker and Crawford note, the “pipeline problem” is often deployed by companies to focus the debate on the small pool of available “diverse” candidates, rather than the “workplace cultures, power asymmetries, harassment, exclusionary hiring practices, unfair compensation, and tokenization” that prevent marginalized people from entering or remaining in STEM sectors (West, Whittaker & Crawford 2019, 3). Moreover, focusing on the *lack* of women and people of color in this industry shifts the burden of responsibility onto “those who are discriminated against, rather than the perpetrators” (West, Whittaker & Crawford 2019, 25). Technosolutionist AI tools, therefore, claim to outsource these difficult HR decisions to technology by selecting workers more efficiently and more “fairly.” However, they fail to address either HR recruiters’ individualized prejudices or the structural forces of sexism and racism that continue to permeate the companies they work for and represent.

The outsourcing of diversity work to technologies, and the subsequent eschewal of responsibility for DEI in hiring, emerges strongly in the marketing materials produced by companies like Retorio. In a piece on how “AI tackles hiring bias,” a representative for Retorio writes:

Whether it’s conscious or unconscious bias, discrimination based on race, gender, disability and many other characteristics continues to distort recruiters hiring decisions. Multiple studies have shown that recruiters [sic] decisions can be unconsciously influenced by their own personal characteristics during the hiring process, as well as societal biases [...] These biases are formed throughout life and held at a subconscious level, as we absorb millions of bits of information that our different brains all interpret in different ways. So, whether we like it or not, some of these deep level biases are almost impossible to mitigate (Briah, 2021a, 2021b, “How AI Tackles Hiring Bias”).

Retorio’s emphasis on how bias inevitably “creeps in” to the hiring process evokes what Rinaldo Walcott calls the “obscuring language” of unconscious bias (Walcott, 2019, 397). Walcott suggests that the language of unconscious bias “refuses to acknowledge how institutions reproduce particular practices and ways of being; and furthermore, it seeks to not blame any particular person or persons for the perpetuation of those practices of advantage making” (Walcott, 2019, 398). Retorio’s passive language of “distortion,” “creep,” and “influenced” evokes this refusal to assume responsibility for the actions and cultures that result in underrepresentation. We are not suggesting that unconscious bias does not exist, but rather that corporations and institutions frequently turn to the language of unconscious bias as a proxy for engaging with the deeply rooted systemic injustices that characterize their workplaces. This outsourcing of diversity work to machines reflects a much longer history of automating undesirable labor; consequently, in the next section, we consider how the history of facial recognition shapes AI recruitment tools’ production of difference (Atanasoski & Vora, 2019).

### Identifying Candidates’ Internal Characteristics

In order to understand how candidates are ascribed value in the hiring process, we must consider how these AI tools play into existing and historical forms of discrimination through their processes of classification. Proponents of AI-powered hiring tools promise that AI-powered facial and body language analysis offers greater and more incisive insight into a candidate’s “authentic” thoughts and feelings. Retorio argues that its software “can analyse candidates speech, gestures and micro-expressions, collect data based on candidates online presence, and assess these large amounts of data to generate personality insights” (Briah, 2021a, 2021b, “Importance of Hiring the Right Personality”). myInterview uses algorithms to similar effect, in order to “interpret body language and automated transcriptions” and “help give Big 5 personality characteristics such as ‘outgoing’ or ‘altruistic’” (myInterview, n.d., “The Definitive Guide,” 5). Moreover, the human resources website *HR Zone* featured an article in 2021 by Chandan Agarwal, COO of global communication software EnableX.io, in which he suggested that Emotion AI can help recruiters “win the talent war” by detecting a candidate’s internal state and emotions more accurately than a human recruiter:

Clearly, gone are the days when a candidate could fake emotions to impress the interviewer since Emotion AI helps the interviewer to detect emotions such as nervousness, distraction, anger, happiness, excitement and more, based on micro-expressions that are not detectable by the naked eye but are highly indicative of the persons [sic] emotional state of mind (enablex, 2021).

This feature in one of the most prominent recruitment publications demonstrates that senior practitioners are buying into the claims made by AI hiring tools regarding how they can recognize and analyze human emotional states at scale and in all contexts. Retorio, for example, insists that its tools are “globally applicable” since they have been trained with a “demographically diverse dataset” of over 100,000 people “from all around the world,” making them especially valuable tools for large, multinational companies and organizations (Retorio, n.d., “The Science Behind Retorio’s Personality Analytics AI”).

However, the characteristics and personality traits that are deemed desirable to employers are neither performed nor interpreted universally and objectively: they are contextual and mediated by racialized and gendered ways of reading the body. The implication of Retorio’s “globally applicable” tools is that algorithms determine innate skills and attributes, and that these traits are expressed in the same way by all bodies, thereby producing a level playing field from which to make assessments. This naturalizes attributes as biological universals, obscuring how they might be learned as well as how they are always culturally contingent. As scholars such as Rinaldo Walcott and Stephanie Smallwoood have argued, the classification and categorization of people is always linked to capital and plays a crucial part in facilitating their exchange on the job market (Walcott, 2019, 402; Smallwood, 2004, 292).

Rather than removing gender and race from their systems altogether, AI-powered tools are part of a much longer lineage of sorting, taxonomizing, and classifying voices and bodies along gendered and racialized lines. Retorio, for example, uses a software called FaceReader Online in conjunction with The Big 5 or Five-Factor model to show “how individual characteristics of thoughts, emotions, and behavior can be mapped in a taxonomy” (Retorio, n.d., “Why Retorio”). When proponents of recruitment AI insist that external appearance is a reliable indicator of internal traits, they give new credence to pseudoscientific ideas that have historically shaped the field of human resources (O’Neil, 2017; Ajunwa, 2021). Techniques and methods rooted in scientific racism were used for the purpose of hiring in the early twentieth century, when Katherine Blackford outlined a character analysis for human resources based on an analysis of candidates’ faces. It has been argued that while this was essentially a combination of phrenology and eugenics that stemmed from much longer histories of racist pseudoscience, it continues to influence what we consider to be an employable subject today (Todorov, 2017; West, Whittaker & Crawford, 2019).

As this history shows us, being in the position of the “observer”—rather than the “observed”—is in and of itself a function of power (Sontag, 2003; Browne, 2015). Put differently, the face does not express inner character, but emerges as “charactered” in the process of observation. This means that recruitment AI is not a neutral tool that reads pre-existing qualities in candidates’ bodies, but rather is directly implicated in creating and sustaining the very traits it is imagined to identify. AI hiring companies assume that visual observations can be made without the observer impacting the values that are attributed, and that the subject of identification is distinguishable from the process of identification. This is a premise that feminist physicist Karen Barad among others has disproved in her analysis of how the observational apparatus used in quantum erasure experiments impacts the “observed” reality, as introduced above (Barad, 2007). This finding is of crucial importance when considering how AI hiring tools observe bodies. The body does not precede social norms—they do not merely “overlay” the body—but emerges with and through normative expectations and value systems. Consequently, observations are imbued with the power hierarchies of the era and the perception of particular skills and attributes in certain faces.

By contextualizing hiring AI in this way, our aim is not to claim that hiring technologies are by nature racist but to warn against the belief that they are neutral, that their use of the Big 5 to differentiate candidates is objective, and that they can effectively map personality traits onto faces. Indeed, as mentioned above, a trial of Retorio’s video AI service in February 2021 by journalists at Bavarian Broadcasting in Munich demonstrated how wearing glasses and a headscarf in the video interview decreased the candidate’s (the actor’s) score for conscientiousness and neuroticism respectively (Fergus, 2021). They also found that “the addition of art or a bookshelf in the background made an Asian test subject seem much more conscientious and significantly less neurotic compared to the same faux applicant in front of a plain background” (Fergus, 2021). While the tests did not prove that the system was biased against racialized candidates, it conclusively showed that ascribing value to a candidate’s self-presentation corresponded tangentially at best with the Big 5. This trial provokes the question of what kinds of red herrings and false associations these AI-powered tools create in their claim to accurately taxonomize and classify applicants.

### Producing the Ideal Candidate

In their classification of candidates, AI-powered HR tools analyze the minutiae of a candidate’s speech and bodily movements in order to measure how closely they respond to the employer’s “ideal employee.” In the case of facial recognition software, the minutiae of head movement, expression, and intonation is tracked and interrogated to gauge whether or not a candidate is the “right fit.” Meanwhile, speech recognition software analyzes vocabulary and word choice to discern whether or not a candidate is truly “passionate” about the job and whether their enthusiasm is genuine. In their attempt to “unsee” race and gender, AI-powered tools may actually be less equipped to understand the candidate’s abilities because they have been trained to “see” and observe from the perspective of the employer, who has previously communicated to the AI tool’s account manager a set of predetermined characteristics and traits that denote “good employees.” As universities start to provide students with opportunities to train themselves to perform better in AI-powered interviews (Burke, 2019; Metz, 2020), the ironies of these systems become clear: they claim to erase both the bias of interviewers and the affectations of interviewees, leaving behind only the raw, authentic capacities and traits of the hireable individual. Yet, these technologies themselves are *producing* the very behaviors they claim to observe as candidates attempt to “win over the algorithms” (Metz, 2020).

Moreover, by searching for the employer’s predetermined traits, AI hiring tools play an active role in producing the ideal employee that they claim to “identify.” This occurs during the algorithmic determination of personality categories in hiring contexts, a process which cites and reproduces social norms about who constitutes an “ideal candidate.” Gender studies, queer theory, and Black feminisms emphasize the replicability of norms through language. In the field of AI, feminist interventions have demonstrated the importance of attending to language in order to create AI for the social good (Bones et al., 2021). The shifting arrangements of gendered and racialized words and images within AI hiring tools’ classification systems constitute what Sylvia Wynter calls a “grammar of regularities” and Hortense Spillers views as a “cultural” grammar (Wynter, 1984, 38; Spillers, 1987). They behave like declensions, which in grammatical terms change according to the arrangement of words around them and the meaning they attempt to convey. Understanding these systems’ interpretation of keywords as operating via grammars of description points towards the cultural contingency of systems which operate according to normative exchanges of value-laden signs and symbols. This, we argue, is what is taking place with the rollout of AI hiring tools, which can replicate at scale the norms of expression, keywords, affect, background, and gesture that affect whether or not a person will be hired.

One prominent example of AI hiring’s reproduction of the normative “ideal candidate” is its consideration of whether a candidate will be a good “culture fit.” Retorio, for example, claims that its tools “can determine person-culture fit and estimate whether a person is more or less likely to thrive within an organizational culture” (Retorio, n.d., “The Science Behind”). However, the concept of “culture fit” has long been used as a euphemism to justify the gendered and racialized exclusivity of organizations. Cultural scholar Stuart Hall explores how people learn to express themselves through the transmission of codes and systems of representation that give them the necessary cultural “know-how” to function as culturally competent subjects. He says that children “unconsciously internalize the codes which allow them to express certain concepts and ideas through their systems of representation—writing, speech, gesture, visualization, and so on—and to interpret ideas which are communicated to them using the same systems” (Hall, 1997, 22). If, as Hall suggests, language and behavior are always culturally codified, then more focus should be given to how recruitment AI operates in relation to social behavioral norms, as well as how interpretations of candidates are made actionable according to the candidate requirements of any particular organization. In this context, Hall’s work suggests that the personality of the candidate always emerges *in relation* to the algorithm’s determination of culture fit (also in relation to a particular company, industry, or job role).

This process is automated by AI when it interprets motions and keywords through culturally contingent behavioral and linguistic norms. In doing so, the system seeks as a “culture fit” a predefined and quantified ideal successful employee (Epstein, 2021). For example, myInterview claims that one of its products, PhraseID, “summarizes the most common keywords across all candidates and allows you to filter based on those. Recruiters can easily see what their candidates have in common. Whether it’s social media trends or common work experiences, recruiters will have a clearer idea of who their future employees are and find the right cultural fit” (myInterview, “myInterview IntelligenceTM,” n.d., 5). As Wendy Chun argues, machine learning models are understood as predictive; however, since they are trained on past data, they are re-iterating decisions made in the past, not the future (Chun, 2021). As Chun notes, “it’s not simply that if the training data is sexist it will make sexist predictions, but they’ll only be validated as correct if they make sexist predictions. And that perpetuation of these predictions as true profoundly shapes what is considered to be true in the future” (Chun, 2021). In these systems, Chun suggests “truth equals repetition”; consequently, the candidate that is considered the “best fit” will be the one who corresponds most closely to the existing workforce (Chun, 2021). Put differently, when PhraseID makes hiring recommendations based on common keywords, personality traits, and culture fit, it is also creating universal normatives. Motions and keywords are therefore graded against a company’s normative expectation of candidate fit and competency.

## Conclusions

In this paper, we contest two key claims offered by recruitment AI companies in relation to the development and deployment of AI-powered HR tools:

1. Recruitment AI can objectively assess candidates by removing gender and race from their systems.
2. This removal of gender and race will make recruitment fairer, help customers attain their DEI goals, and lay the foundations for a truly meritocratic culture to thrive within an organization.

We argued that these claims are misleading for four reasons:

**First**, attempts to “strip” gender and race from AI systems often misunderstand what gender and race are, casting them as isolatable attributes rather than broader systems of power.

**Second**, the attempted outsourcing of “diversity work” to AI-powered hiring tools may entrench cultures of inequality and discrimination by failing to address systemic problems within organizations.

**Third**, AI hiring tools’ supposedly neutral assessment of candidates’ traits belies the power relationship between the observer and the observed. Specifically, the racialized history of character analysis and its associated processes of classification extend histories of taxonomical sorting and reflect the current demands and desires of the job market, even when not explicitly conducted along the lines of gender and race.

**Fourth**, recruitment AI tools help produce the “ideal candidate” that they supposedly identify by constructing associations between words and people’s bodies.

These four arguments show that it is essential for hiring AI firms and their clients to engage with how recruitment AI, like the wider process of recruitment, produces and perpetuates gendered and racialized differences between groups and individuals. While this certainly involves addressing instances of gendered or racialized bias found in specific recruitment AI tools, it also necessitates serious engagement with how gender and race are encoded into systems, and why they cannot be so easily removed, as some debiasing measures would suggest. AI-powered hiring firms’ emphasis on how their tools debias the hiring process deflects from the material inequities and (de)privations that shape applicants’ experiences and profiles. These inequities cannot be so easily prised apart from the way that AI tools perceive candidates’ behavior, expression of personality, use of keywords, and general self-(re)presentation. Anti-bias measures therefore should not replace attempts to support minoritized candidates in the hiring process, nor should they represent an end to racial and gender discrimination. Outsourcing recruitment to “neutral” technologies may be an aspiration for recruiters and AI hiring companies alike, but they are not consistent with strategies which have been proven to be impactful at remedying injustices in hiring: these include, for example, making sure that candidates from different backgrounds are well represented in assessment centers by examiners on the basis of race and gender, as well as other interrelated axes of social differentiation such as age and ability (Solicitors Regulation Authority, 2020, 16). Here, feeling understood and fitting in *on the basis of* personal characteristics rather than because of their removal gave candidates the opportunity to perform at their best and get the job.

From the four conclusions outlined above, we offer three key recommendations that speak to the complex network of actors that support the growth and delivery of HR AI products, including the HR AI companies themselves, their customers, policymakers, regulators, and AI ethicists. We hope that the breadth of our approach encourages these actors to pursue what these recommendations mean for them practically and apply them to their specific contexts.

## Recommendations

First, industry practitioners developing hiring AI technologies must shift from trying to correct individualized instances of “bias” to considering the broader inequalities that shape recruitment processes. Pratyusha Kalluri argues that AI experts should not focus on whether or not their technologies are technically fair but whether they are “shifting power” towards the marginalized (Kalluri, 2020). This requires abandoning the “veneer of objectivity” that is grafted onto AI systems (Benjamin, 2019a, 2019b) so that technologists can better understand their implication—and that of the corporations within which they work—in the hiring process. For example, practitioners should engage with how the categories being used to sort, process, and categorize candidates may have historically harmed the individuals captured within them. They can then begin to problematize the assumptions about “gender” and “race” they are building into AI hiring tools even as they intend to strip racial and gender attributes out of recruitment.

Second, we suggest that HR practitioners who are purchasing and using these tools must engage meaningfully with the ways that AI is shifting power in their own field. Time-pressed and overworked HR professionals are understandably excited by the potential of AI to revolutionize HR practices. However, while recognizing the extra demands this places on HR professionals, we believe it is essential that HR professionals possess at the very least a basic understanding of the limitations of AI-powered hiring tools. Again, this requires lifting the veil of objectivity from hiring AI and critically scrutinizing AI companies’ claims that their technologies will magically solve HR departments’ long-term struggles around both volume recruitment and their DEI agendas. It also requires HR professionals to compel third-party vendors to disclose exactly where AI is being used in their systems and how it is being used to evaluate candidates. Only through this heightened awareness of the AI capabilities of new HR tools can the field of HR seriously grapple with both the benefits and the risks posed by new and emerging AI technologies.

Third, while it is encouraging to see greater scrutiny and legislation in relation to AI-powered HR tools, there is still largely an insufficient contribution from AI ethicists, regulators, and policymakers. Serious concerns also remain in regard to the efficacy of these regulatory approaches. For example, the Artificial Intelligence Video Interview Act (2020) only covers AI used in video hiring tools, meaning that AI used in other parts of the hiring process (such as CV sifting or speech recognition) remains unaddressed. Additionally, as suggested by technology policy non-profit Upturn in their report on hiring technologies, candidates should be informed when they are being processed and evaluated by AI software, and understand the technology well enough to be able to reject its use and propose an alternative form of recruitment if required (Bogen & Rieke, 2018, 12). Since AI-powered HR tools can and will reproduce forms of difference and discrimination, it is crucial that the AI ethics community scrutinize the claims and the products made by AI hiring companies, and how these technologies are deployed in industry contexts. Specifically, it must combat companies’ claim that these technologies remove any evidence of “difference,” and instead interrogate how these supposedly “neutral” technologies are themselves actively involved in the production and inscription of difference throughout the hiring process.

## Data Availability

All the materials that we discussed in this paper are publicly available online.

## Footnotes

1 For exceptions, see O’Neil (2017) and Ajunwa (2021).

2 The “Big 5” traits are Extraversion, Agreeableness, Conscientiousness, Emotional Stability, and Intellect. Although the Big 5 can be traced back to psycho-lexical approaches to personality that emerged in the latter half of the ninetennth century, contemporary iterations of the Big 5 model are often attributed to Lewis Goldberg (1990) and Paul Costa and Robert McCrae’s Five-Factor model (FFM) from 1992.

## References

[CR1] *biased misconceptions of the hiring process and what you can do to mitigate them*. (2019, July 23). Blog.Myinterview. Retrieved March 21, 2022 from https://blog.myinterview.com/worried-about-bias-in-your-hiring-process.

[CR2] Ahmed, S. (2012). *On being included: Racism and diversity in institutional life*. Duke University Press.

[CR3] Ajunwa, I. (2021) Automated video interviewing as the new phrenology. *Berkeley Technology Law Journal* 36, 102–152. https://ssrn.com/abstract=3889454

[CR4] Albert, E. T. (2019). AI in talent acquisition: A review of ai-applications used in recruitment and selection. *Strategic HR Review, 18*(5), 215–221.

[CR5] Alexander, M. J. (2005). *Pedagogies of crossing: Meditations on feminism, sexual politics, memory, and the sacred*. Duke University Press.

[CR6] Amoore L (2020). Cloud ethics: Algorithms and the attributes of ourselves and others.

[CR7] Anderson, M. & Perrin, A. (2017, May 17) *Barriers to adoption and attitudes towards technology*. Pew Research Center. Retrieved March 21, 2022 from https://www.pewresearch.org/internet/2017/05/17/barriers-to-adoption-and-attitudes-towards-technology/

[CR8] ASA. (n.d.). *03 Misleading advertising: CAP Code*. Asa.Org.Uk. Retrieved January 21, 2022, from https://www.asa.org.uk/type/non_broadcast/code_section/03.html

[CR9] Atanasoski N, Vora K (2019). Surrogate humanity: Race, robots, and the politics of technological futures.

[CR10] Bankins S (2021). The ethical use of artificial intelligence in human resource management: A decision-making framework. Ethics and Information Technology.

[CR11] Barad K (2007). Meeting the universe halfway: Quantum physics and the entanglement of matter and meaning.

[CR12] Benjamin R (2019). Race after technology: Abolitionist tools for the new jim code.

[CR13] Benjamin R (2019). Captivating technology: Race, carceral technoscience, and liberatory imagination in everyday life.

[CR14] Benjamin, M., Buehler, K., Dooley, R., & Zipparo, P. (2021, August 12). *What the draft European Union AI regulations mean for business*. McKinsey & Company. Retrieved January 21, 2022, from https://www.mckinsey.com/business-functions/mckinsey-analytics/our-insights/what-the-draft-european-union-ai-regulations-mean-for-business

[CR15] Bogen, M., & Rieke, A. (2018, December). *Help wanted: An examination of hiring algorithms, equity, and bias.* Upturn.org. Retrieved March 21, 2022 from https://www.upturn.org/static/reports/2018/hiring-algorithms/files/Upturn%20--%20Help%20Wanted%20-%20An%20Exploration%20of%20Hiring%20Algorithms,%20Equity%20and%20Bias.pdf

[CR16] Bones, H., Ford, S., Hendery, R., Richards, K. & Swist, T. (2021) In the frame: The language of AI. *Philosophy & Technology* 34l: 23–44. 10.1007/s13347-020-00422-7

[CR17] Briah (2021, August 6). *Importance of hiring the right personality - and how AI can help.* Retorio.com. Retrieved March 21, 2022 from https://www.retorio.com/blog/importance-of-hiring-the-right-personality-and-how-ai-can-help#:~:text=AI%20can%20analyse%20candidates%20speech,data%20to%20generate%20personality%20insights.

[CR18] Briah (2021a, July 26). *How AI is revolutionising the recruitment process*. Retorio.com. Retrieved March 22, 2022 from https://www.retorio.com/blog/how-ai-is-revolutionising-the-recruitment-process

[CR19] Briah (2021b, August 2). *How AI tackles hiring bias.* Retorio.com. Retrieved March 21, 2022 from https://www.retorio.com/blog/how-ai-tackles-hiring-bias

[CR20] Browne, S. (2015) *Dark Matters: On the Surveillance of Blackness. *Durham: Duke University Press.

[CR21] Butler J (2011). Bodies that matter: On the discursive limits of sex.

[CR22] Burke, L. (2019) Your interview with AI. *Inside higher* Ed, Nov 4. Retrieved 20/6/2022 from https://www.insidehighered.com/news/2019/11/04/ai-assessed-job-interviewing-grows-colleges-try-prepare-students

[CR23] Caldwell JT (2008). Production culture: Industrial reflexivity and critical practice in film and television.

[CR24] Cappelli, P. (2021, December 7). *Cappelli: Complexities of employee monitoring in an AI age*. HR Executive. Retrieved January 21, 2022, from https://hrexecutive.com/why-ai-is-a-talent-management-enabler/

[CR25] Carlyle. (n.d.). *HireVue*. Retrieved January 21, 2022, from https://www.carlyle.com/impact/hirevue

[CR26] Cave S, Dihal K (2020). The whiteness of AI. Philosophy & Technology.

[CR27] Chun, W. H. K. (2021). Wendy Hui Kyong Chun on facebook ‘friendship’ and predicting the future. *The Good Robot* https://feeds.buzzsprout.com/1786427.rss

[CR28] D’Ignazio C, Klein LF (2020). Data feminism.

[CR29] Danks, D., & London, A. J. (2017). Algorithmic bias in autonomous systems. *IJCAI International Joint Conference on Artificial Intelligence*, 4691–4697. 10.24963/ijcai.2017/654

[CR30] Dastin, J. (2018, October 11). *Amazon scraps secret AI recruiting tool that showed bias against women*. U.S. Retrieved January 21, 2022, from https://www.reuters.com/article/us-amazon-com-jobs-automation-insight-idUSKCN1MK08G

[CR31] Dihal K, Cave S, Dihal K, Dillon S (2020). Enslaved minds: Artificial intelligence, slavery and revolt. AI narratives: A history of imaginative thinking about intelligent machines.

[CR32] Drage, E., & Frabetti, F. (forthcoming). AI that matters: A feminist approach to the study of intelligent machines. In Browne, J., Cave, S., Drage, E., & Mackereth, K. (Ed.), *Feminist AI: Critial perspectives on algorithms, data and intelligent machines* (pp. 1–3). Oxford: Oxford University Press.

[CR33] Durose, C., Gains, F., Richardson, L., Combs, R., Broome, K., Eason, C. (2011). Pathways to politics. Equality and Human Rights Commission. Retrieved 21 March, 2022 from https://www.equalityhumanrights.com/sites/default/files/research-report-65-pathways-to-politics.pdf

[CR34] Edwards, M.; Mason, B.; Bajorek, Z.; Holmes, J.; & Lucy, D. (2021) *Good recruitment for older workers: The current and future recruitment landscape*. Centre for Ageing Better. Retrieved March 21, 2022 from https://ageing-better.org.uk/sites/default/files/2021-01/Good-recruitment-for-older-workers.pdf

[CR35] Elizabeth T. (2020, February 17). *The ‘Amazon’ candidate experience: Millennials and gen Z want AI*. Retorio. Retrieved March 21, 2022 from https://www.retorio.com/blog/-candidate-experience-millennials-gen-z-ai.

[CR36] Epstein, S. (2021, October 20). *What does being a “cultural fit” actually mean?* BBC Worklife. Retrieved January 21, 2022, from https://www.bbc.com/worklife/article/20211015-what-does-being-a-cultural-fit-actually-mean

[CR37] *EUR-Lex - 52021PC0206 - EN - EUR-Lex*. (2021, April 21). Eur-Lex.Europa.Eu. Retrieved March 21, 2022 from https://eur-lex.europa.eu/legal-content/EN/TXT/?uri=CELEX%3A52021PC0206

[CR38] Feffer, M. (2020, September 16) *Top 10 video interviewing tools for a virtual hiring toolkit.* Tech Target. Retrieved March 14, 2022 from https://www.techtarget.com/searchhrsoftware/feature/Top-10-video-interviewing-tools-for-a-virtual-hiring-toolkit

[CR39] Fergus, J. (2021, February 18). *A bookshelf in your job screening video makes you more hirable to AI*. Input. Retrieved March 21, 2022 from https://www.inputmag.com/culture/a-bookshelf-in-your-job-screening-video-makes-you-more-hirable-to-ai.

[CR40] Ferguson AG (2017). The rise of big data policing: Surveillance, race, and the future of law enforcement.

[CR41] Fritts M, Cabrera F (2021). AI recruitment algorithms and the dehumanization problem. Ethics and Information Technology.

[CR42] Futurity Media. (2020, February 12). *How NOT to write a white paper*. Retrieved January 21, 2022, from https://www.futuritymedia.com/blog/how-not-to-write-a-white-paper/

[CR43] Gartner. (2020, April 30). Virtual interviews to hire candidates during COVID. Retrieved March 21, 2022 from https://www.gartner.com/en/newsroom/press-releases/2020-04-30-gartner-hr-survey-shows-86--of-organizations-are-cond

[CR44] Gilroy, P. (2009, December). *Race and the right to be human*. Inaugural lecture delivered on December 3, 2009 on the occasion of accepting the Treaty of Utrecht Chair at Utrecht University, Utrecht. Retrieved March 21, 2022 from https://www.uu.nl/sites/default/files/gw_gilroy_paul_oratie_definitief.pdf

[CR45] Guerrero, N. (2021) *What is anonymous mode?* Censia, Nov 16. Retrieved 21/6/2022 from https://www.censia.com/blog/whatis-anonymous-mode/

[CR46] Gumbus A, Grodzinsky F (2004). Gender bias in internet employment: A study of career advancement opportunities for women in the field of ICT. Journal of Information, Communication and Ethics in Society.

[CR47] Hall, S. (1997). *Representation: Cultural representations and signifying practices (culture, media and identities series)* (1st ed.). Sage Publications & Open University.

[CR48] Hao, K. (2021, June 17). *Facebook’s ad algorithms are still excluding women from seeing jobs*. MIT Technology Review. Retrieved January 21, 2022, from https://www.technologyreview.com/2021/04/09/1022217/facebook-ad-algorithm-sex-discrimination/

[CR49] Heilweil, R. (2020, January 1). Illinois regulates artificial intelligence like HireVue’s used to analyze online job Interviews. Vox. https://www.vox.com/recode/2020/1/1/21043000/artificial-intelligence-job-applications-illinios-video-interivew-act

[CR50] Heinrichs, B. (2021). Discrimination in the age of artificial intelligence. AI and Society, 0123456789. 10.1007/s00146-021-01192-2

[CR51] HireVue. (n.d.). *Train, Validate, Re-train*. Hirevue.Com. https://www.HireVue.com/blog/hiring/train-validate-re-train-how-we-build-HireVue-assessments-models

[CR52] HireVue. (n.d.). *HireVue + Unilever: Unilever + HireVue Unilever finds top talent faster with HireVue assessments*. Hirevue.Com. Retrieved January 21, 2022d, from https://www.hirevue.com/case-studies/global-talent-acquisition-unilever-case-study

[CR53] HireVue. (n.d.) *Frequently asked questions*. HireVue.Com. Retrieved March 21, 2022 from https://www.hirevue.com/candidates/faq.

[CR54] HireVue. (n.d.). *Case studies: The co-operative bank*. HireVue.Com Retrieved January 21, 2022a, from https://www.HireVue.com/case-studies/co-operative-bank

[CR55] HireVue. (n.d.). *Increase diversity and mitigate bias*. Hirevue.Com. Retrieved March 22, 2022b from https://www.hirevue.com/employment-diversity-bias

[CR56] HireVue. (n.d.). *Maggiano’s Little Italy + HireVue*. Hirevue.Com. Retrieved March 22, 2022c from https://www.HireVue.com/case-studies/maggianos-little-italy

[CR57] HireVue (n.d.) Unlike most things. In *2020, hiring is not cancelled*!. Retrieved 21/06/2022 from https://v7w4r4b5.stackpathcdn.com/wp-content/uploads/2020/08/hiring-is-not-cancelled-infographic.pdf

[CR58] Hooks B (1992). Black looks: Race and representation.

[CR59] enablex. (2021, June 9). *How Emotion AI could help you win the talent war*. Retrieved January 21, 2022, from https://www.hrzone.com/community/blogs/enablex/how-emotion-ai-could-help-you-win-the-talent-war

[CR60] Illinois General Assembly. (2020, January 1). *820 ILCS 42/ Artificial Intelligence Video Interview Act. Ilga.Gov.* Retrieved March 22, 2022 from https://www.ilga.gov/legislation/ilcs/ilcs3.asp?ActID=4015&ChapterID=68

[CR61] Inside Higher Ed. (2019, November 4). *As AI-assessed job interviewing grows, colleges try to prepare*. Retrieved January 21, 2022, from https://www.insidehighered.com/news/2019/11/04/ai-assessed-job-interviewing-grows-colleges-try-prepare-students

[CR62] Interviewer.ai. (n.d.) *Explainable AI*. Retrieved March 1, 2022, from https://interviewer.ai/explainable-ai/

[CR63] Jasanoff, S., & Kim, S.-H. (2009). Containing the atom: Sociotechnical imaginaries and nuclear power in the United States and South Korea. *Minerva, 47*(2), 119–146 http://www.jstor.org/stable/41821489

[CR64] Kalluri, P. (2020). *Don’t ask if artificial intelligence is good or fair, ask how it shifts power*. Nature. Retrieved March 14, 2022 from https://www.nature.com/articles/d41586-020-02003-2?utm_source=twt_nnc&utm_medium=social&utm_campaign=naturenews&error=cookies_not_supported&code=bcb71409-8168-4ee3-98f5-8e7578698393 10.1038/d41586-020-02003-2 32636520

[CR65] Kane, L. (2019, September 8) *Usability for seniors: Challenges and changes*. Nielsen Norman Group. Retrieved March 21, 2022 from https://www.nngroup.com/articles/usability-for-senior-citizens/

[CR66] King-O’Riain RC, Small S, Song M, Spickard P, Mahtani M (2014). Global mixed race. Global mixed race.

[CR67] Knight, W. (2021, January 12). *Job screening service halts facial analysis of applicants*. Wired. https://www.wired.com/story/job-screening-service-halts-facial-analysis-applicants/

[CR68] Kontzer, T. (2018, May 18) *Huge potential of video interview software comes with risks*. TechTarget. Retrieved March 21, 2022 from https://www.techtarget.com/searchhrsoftware/feature/Huge-potential-of-video-interview-software-comes-with-risks?amp=1

[CR69] Lewis, J. E. (2021, May 28) *From impoverished intelligence to abundant intelligences.* Medium. Retrieved March 22, 2022 from https://jasonedwardlewis.medium.com/from-impoverished-intelligence-to-abundant-intelligences-90559f718e7f.

[CR70] Lin YT, Hung TW, Huang LTL (2021). Engineering equity: How AI can help reduce the harm of implicit bias. Philosophy and Technology.

[CR71] Mahtani M (2014). Mixed race amnesia: Resisting the romanticization of multiraciality.

[CR72] Metz, R. (2020) *There’s a new obstacle to landing a job after college: Getting approved by AI*. CNN Business, Jan 15. Retrieved 20/6/2022 from https://edition.cnn.com/2020/01/15/tech/ai-job-interview/index.html

[CR73] Mohamed S, Png M-T, Isaac W (2020). Decolonial AI: Decolonial theory as sociotechnical foresight in artificial intelligence. Philosophy and Technology.

[CR74] Mohanty, C. T. (2003). *Feminism without borders: Decolonizing theory, practicing solidarity*. Duke University Press.

[CR75] Myers West, S., Whittaker, M., & Crawford, C. (2019, April). *Discriminating systems: Gender, race, and power in AI.* AI Now. Retrieved March 22, 2022 from https://ainowinstitute.org/discriminatingsystems.pdf

[CR76] myInterview (n.d.). myInterview: Intelligent candidate video screening. MyInterview. Retrieved March 22, 2022 from https://www.myinterview.com/product-intelligence/

[CR77] myInterview. (n.d.). *The definitive guide to AI for human resources.* myInterview.Com. Retrieved March 22, 2022 from https://Explore.Myinterview.Com/Myinterviewintelligence.

[CR78] myInterview. (n.d). *myInterview IntelligenceTM: How AI is paving the way for faster and more effective hiring.* myInterview.Com. Available sent to email on request from the chatbot at myInterview.com.

[CR79] myInterview. (n.d.). MyInterview. Retrieved March 22, 2022 from https://www.myinterview.com/

[CR80] myInterview. (n.d.). *myInterview Intelligence*. MyInterview.Com. Retrieved March 22, 2022 from https://explore.myinterview.com/myinterviewintelligence

[CR81] Newkirk, P. (2019). *Diversity Inc: The failed promise of a billion-dollar business*. Bold Type Books.

[CR82] Noble S (2018). Algorithms of oppression: How search engines reinforce racism.

[CR83] O’Neil C (2017). Weapons of math destruction: How big data increases inequality and threatens democracy.

[CR84] Popejoy, A. B. (2021, August 24) Too many scientists still say Caucasian*. Nature World View*. Retrieved Marcy 14, 2022 from https://www.nature.com/articles/d41586-021-02288-x

[CR85] PWC. (2019, April). *Women in Tech: Time to close the gender gap*. Retrieved March 22, 2022 from https://www.pwc.co.uk/women-in-technology/women-in-tech-report.pdf

[CR86] Rachel Metz, CNN Business. (2020, January 15). *There’s a new obstacle to landing a job after college: Getting approved by AI*. CNN. Retrieved March 22, 2022 from https://edition.cnn.com/2020/01/15/tech/ai-job-interview/index.html

[CR87] Retorio (n.d.) *Big Five*. Retorio.Com. Retrieved March 22, 2022 from ​​https://www.retorio.com/retorio-big-five-overview

[CR88] Retorio. (n.d.). *Why Retorio*. Retorio.Com. Retrieved March 22, 2022 from https://www.retorio.com/science

[CR89] Retorio. (n.d.). Personality Assessment. Retorio.com. Retrieved March 22, 2022a from https://www.retorio.com/personality-assessment

[CR90] Retorio. (n.d.). *Retorio | Whitepaper*. Retorio.Com. Retrieved January 21, 2022b, from https://www.retorio.com/whitepaper/download-0-0-0?submissionGuid=5e6130c9-b9bc-4de5-acc4-dc757a7558fe

[CR91] Retorio. (n.d.). *The science behind Retorio’s AI-powered personality test*. Retorio.Com. Retrieved March 22, 2022c from https://www.retorio.com/aiscience.

[CR92] Roth PL, Huffcutt AI (2013). A meta-analysis of interviews and cognitive ability. Journal of Personnel Psychology.

[CR93] Sage. (2020). *The changing face of HR: Research report 36: HR and People Leaders’ Report*. Retrieved March 22, 2022 from https://online.sageintacct.com/rs/473-QSL-641/images/changing-face-hr-research-HR-People-Mgt.pdf

[CR94] Schellmann, H., & Wall, S. (2021, July 13). *We tested AI interview tools. Here’s what we found.* MIT Technology Review. Retrieved March 22, 2022 from https://www.technologyreview.com/2021/07/07/1027916/we-tested-ai-interview-tools/

[CR95] Schwerzmann K (2021). Abolish! Against the use of risk assessment algorithms at sentencing in the US Criminal Justice System. Philosophy and Technology.

[CR96] Smallwood, S. (2004). Commodified freedom: Interrogating the limits of anti-slavery ideology in the Early Republic. *Journal of the Early Republic*, *24*(2), 289–298. https://www.jstor.org/stable/4141508

[CR97] Sontag, S. (2003). *Regarding the pain of others*. Hamish Hamilton.

[CR98] Solicitors Regulation Authority. (2020, June). Implementing the Solicitors Qualifying Examination (SQE). SRA.org. https://www.sra.org.uk/globalassets/documents/sra/board-meetings/2020/sra-board-item-sqe-implementation-complete.pdf?version=49c663

[CR99] Spillers HJ (1987). Mama’s baby, papa’s maybe: An American grammar book. Diacritics.

[CR100] Strengers Y, Kennedy J (2020). The smart wife: Why Siri, Alexa, and other smart home devices need a feminist reboot.

[CR101] Tilmes, N. (2022). Disability, fairness, and algorithmic bias in AI recruitment. *Ethics of Information Technology 24*, 21. 10.1007/s10676-022-09633-2

[CR102] The University of Arizona. (n.d.). *Writing a white paper | UAGC Writing Center*. Writingcenter.Uagc.Edu. Retrieved January 21, 2022, from https://writingcenter.uagc.edu/writing-white-paper

[CR103] Thomé, J. (2021, December 30). *How talent intelligence removes bias and increases diversity in recruiting*. Censia. Retrieved March 22, 2022 from https://www.censia.com/blog/how-talent-intelligence-increases-diversity/

[CR104] Todorov A (2017). Face value: The irresistible influence of first impressions.

[CR105] Tussey E (2018). The procrastination economy: The big business of downtime.

[CR106] van den Broek, E., Sergeeva, A., & Huysman, M. (2019). Hiring algorithms: An Ethnography of Fairness in Practice. ICIS 2019 Proceedings. 6. https://aisel.aisnet.org/icis2019/future_of_work/future_work/6

[CR107] Vogels, E. A. (2019, September 9). *Millennials stand out for their technology use, but older generations also embrace digital life*. Retrieved March 22, 2022 from https://www.pewresearch.org/fact-tank/2019/09/09/us-generations-technology-use/

[CR108] Walcott, R. (2019). The end of diversity. *Public Culture, 31*(2), 393–408 https://doi-org.ezp.lib.cam.ac.uk/10.1215/08992363-7286885

[CR109] Wang, J. (2018) *Carceral capitalism*. Los Angeles: Semiotext(s).

[CR110] Weber, J., & Bath, C. (2007). “Social” robots & “emotional” software agents: Gendering processes and de-gendering strategies for “technologies in the making”. In *Gender designs IT: Construction and deconstruction of information society technology* (pp. 53–63). VS Verlag für Sozialwissenschaften. 10.1007/978-3-531-90295-1_3

[CR111] Wellner G, Rothman T (2020). Feminist AI: Can we expect our AI systems to become feminist?. Philosophy and Technology.

[CR112] West, S. M, Whittaker, M. and Crawford, K. (2019) *Discriminating systems: Gender, race and power in AI*. AI Now Institute. Retrieved from https://ainowinstitute.org/discriminatingsystems.html

[CR113] Whittaker, M, Crawford, K, Dobbe, R, et al. (2018). *AI Now Report 2018*. AI Now. Retrieved March 22, 2022 from ainowinstitute.org/AI_Now_2018_Report.pdf

[CR114] Wynter, S. (1984). The ceremony must be found: After humanism. *Boundary 2*, *12*(3), 19–70. 10.2307/302808

[CR115] Yam J, Skorburg JA (2021). From human resources to human rights: Impact assessments for hiring algorithms. Ethics and Information Technology.

[CR116] Yao X (2021). Disaffected: The cultural politics of unfeeling in nineteenth-century America.

[CR117] Young, E., Wajcman, J. and Sprejer, L. (2021). Where are the Women? Mapping the Gender Job Gap in AI. Policy Briefing: Full Report. *The Alan Turing Institute*. Retrieved March 22, 2022 from https://www.turing.ac.uk/sites/default/files/2021-03/where-are-the-women_public-policy_full-report.pdf

